# Stressful Life Events and the Clinical Expression of Obsessive–Compulsive Disorder (OCD): An Exploratory Study

**DOI:** 10.3390/jcm9103371

**Published:** 2020-10-21

**Authors:** André Kracker Imthon, César Antônio Caldart, Maria Conceição do Rosário, Leonardo F. Fontenelle, Euripedes Constantino Miguel, Ygor Arzeno Ferrão

**Affiliations:** 1 Psychiatric Service, President Vargas Hospital, Porto Alegre 90035-074, Brazil; cesar_antoniocaldart@hotmail.com (C.A.C.); ygoraf@gmail.com (Y.A.F.); 2 Department of Internal Medicine-Psychiatry, Federal University of Health Sciences of Porto Alegre, Porto Alegre 90050-170, Brazil; 3 The Brazilian Research Consortium on Obsessive–Compulsive Spectrum Disorders, São Paulo 05403-903, Brazil; mariaceica.rosario@gmail.com (M.C.d.R.); leo.fontenelle@monash.edu (L.F.F.); ecmiguel7@gmail.com (E.C.M.); 4 Child and Adolescent Psychiatry Unit (UPIA) at the Department of Psychiatry, Federal University of São Paulo, São Paulo 04017-030, Brazil; 5 Turner Institute for Brain and Mental Health, Monash University, Clayton VIC 3800, Australia; 6 D’Or Institute for Research and Education (IDOR) and Institute of Psychiatry (IPUB), Federal University of Rio de Janeiro, Rio de Janeiro 22290-140, Brazil; 7 Department of Psychiatry, São Paulo University Medical School, São Paulo 05403-903, Brazil

**Keywords:** obsessive–compulsive symptoms, obsessive–compulsive disorder, stressful life events, symptom dimensions, risk factors

## Abstract

Background: In obsessive–compulsive disorder (OCD), symptom content and severity appear to fluctuate over the course of the life cycle in accordance with stressful life events. The objective of this paper was to compare OCD patients with and without reported stressful life events (SLEs) in terms of the sociodemographics of patients and the clinical characteristics of OCD. Methods: This was a cross-sectional study involving 1001 patients with OCD. Data concerning SLEs were collected via the Yale OCD Natural History Questionnaire, while for OCD symptoms, the Dimensional Yale–Brown Obsessive–Compulsive Scale was used. Results: Of the 1001 OCD patients, 605 (60.5%) reported experiencing at least one SLE in their lifetime. Self-declared nonwhite skin color (odds ratio (OR) = 1.51), the presence of a sensory phenomenon (OR = 1.47), and comorbidity with post-traumatic stress disorder (PTSD) (OR = 2.38) were some of the logistic regression variables related to the reported SLEs with relevant statistical significance and risk (i.e., OR) values. Conclusions: Our results indicate that SLEs may make Brazilian OCD patients vulnerable to the onset or exacerbation of obsessive–compulsive symptoms. The positive association of the occurrence of SLEs and sensory phenomena in this population could corroborate that environmental influences impact the neurobiology associated with OCD, and likely with other psychiatric disorders as well.

## 1. Introduction

Obsessive–compulsive disorder (OCD) is a heterogeneous condition, composed of distinct subtypes [1]. These subtypes can be described according to the predominant obsessions or compulsions, the age at onset [2,3,4,5,6], the content of the obsessions or compulsions [7,8,9], insight level [10,11], the presence of sensory phenomena [12,13], the presence of tics [14,15,16,17], psychiatric comorbidities [18,19], the response to conventional treatments [20,21,22], the presence of a familial history of OCD [23,24,25], and many other clinical features. The relationship between environmental factors and each of these subtypes or the course or severity of the clinical features of OCD, however, has not yet been substantially explored. 

It is known that environmental factors are very important factors that impact the likelihood of obtaining, persisting, or remitting with respect to obsessive–compulsive symptoms (OCS) [26]. For example, some studies have shown that sexual abuse appears to be an important mediator of subsequent onset of OCD [26,27]. Stressful life events (SLEs) are defined as a combination of major life events and the subjective perception of the relevance of these events by the individual [28], and they are recognized to contribute to the onset or worsening of symptoms of diverse major psychiatric disorders, as explained by the classic diathesis–stress model [29]. It is known that SLEs play a crucial role in the onset of many psychiatric disorders, such as major depression [30,31,32,33], and in trauma- and stress-related disorders [34,35]. Adams et al. [36] postulated that it is unclear whether trauma or SL Escause OCD, are triggering factors that interact with pre-existing vulnerabilities, or are simply nonspecific factors that can exacerbate OCD along with other aspects of psychiatric symptomatology. 

The impact of environmental stress and/or SLEs on OCD has been explored by some authors. Maina et al. [37] explored the occurrence of SLEs in a small sample of OCD patients and found a nonsignificant excess of SLEs in patients compared to that in healthy subjects. Fontenelle et al. [38] explored the influence of traumatic events on the pattern of the clinical presentation of OCD and noted that it may modulate the onset and the content of the symptoms, leading patients with post-traumatic OCD onset to show later onset and to present contamination/cleaning/washing symptoms more frequently than patients with pre-traumatic onset did [38]. Most of the cited papers investigated SLEs in relatively small OCD patient samples, exploring OCD severity as a homogeneous disorder and the occurrence of specific OCS as a categorical variable, but they did not include specific information (such as sensory phenomena, level of insight, suicidality, psychiatric comorbidities, and the therapeutic strategies used) and have not yet investigated the occurrence of SLEs in a Latin sample of OCD patients.

The present exploratory study aimed to compare patients with and without reported SLE swith regard to the severity and expression of specific dimensions of OCD symptoms (according to the Dimensional Yale–Brown Obsessive–Compulsive Scale (DY-BOCS)) as well as other psychopathological features (i.e., gender, age at onset, OCD familial history, OCD course, presence of sensory phenomena, insight into OCS, severity of depressive and anxious comorbid symptoms, occurrence of specific lifetime psychiatric comorbidities, and employed treatment options) in a large Brazilian sample of OCD patients. We hypothesize that the severity of OCD symptoms will be higher and that there will be a higher prevalence of aggressive and sexual/religious dimensions, as well as a higher severity and presence of major depression, other anxiety disorders, and suicidality in the SLE group.

## 2. Experimental Section

### 2.1. Methodology

This study is part of the Brazilian Research Consortium on Obsessive–Compulsive Spectrum Disorders (CTOC), which is a multicentric project involving seven research centers located in diverse regions of Brazil [39]. This was a cross-sectional study, with data from 1001 patients who had a primary diagnosis of OCD. All of the patients were evaluated between 2003 and 2008 via clinical interviews and questionnaires. More information regarding the methodology of the data acquisition process is described in another study [39]. The primary OCD diagnosis of these patients was confirmed by the Structured Clinical Interview for DSM-IV Axis I Disorders (SCID-I) [40,41]. We obtained written consent from all of the participants included in this study according to the principles established in the Declaration of Helsinki. All research conducted by the CTOC is approved by the local research ethics committees of the centers involved prior to commencement. 

The “Initial Evaluation Protocol” of the CTOC was used to assess all participants. This protocol is an assessment package (available in Brazilian Portuguese upon request) designed to collect medical history, socioeconomic, and sociodemographic data. Furthermore, it includes a semi-structured interview regarding familial history of psychiatric disorders, standardized scales, questionnaires, and inventories. Questions assessing personal and familial history of suicidal ideation or behavior are also included. The other main instruments utilized in this study were as follows:

Yale–Brown Obsessive–Compulsive Scale (Y-BOCS) [42,43]—one of the main scales used worldwide in order to evaluate the severity of OCS.

- Dimensional Yale–Brown Obsessive–Compulsive Scale (DY-BOCS) [44]—the DY-BOCS assesses OCS by specific dimensions, allowing the severity of each dimension to be independently quantified. This scale has some advantages, for example, avoidant behaviors, as well as rituals (physical or mental), are investigated within each dimension. As a result of this more detailed evaluation process, this scale provides a more precise evaluation of symptom severity. The DY-BOCS evaluates the time spent on OCS, as well as the level of anxiety and the impact of the symptoms, with scores ranging from 0 to 5 with a maximum of 15 for each dimension. The negative impact of OCS on the lives of subjects is also quantified on a scale, with a maximum score of 15. Combining the two scores mentioned before, the maximum total DY-BOCS score is 30.
- Yale OCD Natural History Questionnaire [45]—this questionnaire consists of a comprehensive semi-structured interview. This questionnaire focuses on the course and onset of OCS, relating to life events and situations. It addresses whether specific life events could trigger, worsen, or improve OCS. Part I of this questionnaire addresses OCS onset, exploring the age at onset, whether the symptoms began abruptly or gradually, and whether they started after personal problems, emotional problems, drug use, family problems, financial problems, or the onset of a clinical disease. Part II evaluates the course of OCS and includes questions regarding environmental factors that could affect OCS (e.g., mood, fatigue, sleep, alcohol, coffee, tobacco, certain kinds of food, and weather). These factors are organized in a table where the patient may answer “no effect,” “worse OCS,” “improved OCS,” or “nonapplicable” (a specific paper on these aspects is in preparation); a second table is also provided that indicates the severity of OCS according to the age of the patient. Part III investigates the period with the most OCS, comprising questions similar to those mentioned above but specifically related to the worst period regarding symptom presentation. In the present study, Part I questions were used. To each of the questions, there are two possible responses: “yes” and “no.” When the response was “yes,” complementary questions were asked (e.g., “If yes, describe it better” or “If yes, choose one example option listed below”). If the answer to these questions was considered satisfactory by the interviewer, the participant was assigned to the “SLE” group.
- Structured Clinical Interview for Diagnosis of Axis I Psychiatric Disorders (SCID-I) [40,41]—additional modules evaluating tic and impulse-control disorders were administered [41]. All DSM-IV axis I disorders, except for schizophrenia, which was an exclusion criterion, were included in the analysis. Attention-deficit/hyperactivity disorder (ADHD) and separation anxiety disorder were assessed utilizing the Kiddie Schedule for Affective Disorders and Schizophrenia [46].
- Beck Depression Inventory (BDI) (21 items) and Beck Anxiety Inventory (BAI) [47,48]—both scales were extensively used in order to determine the severity of the symptoms of depression and anxiety.
- The University of São Paulo Sensory Phenomena Scale (USP-SPS) [49]—this is a semi-structured scale designed to investigate the presence and severity of different types of sensory phenomena occurring before or during the performance of repetitive behaviors. The USP-SPS is divided into two parts: achecklist (including different types of sensory phenomena, i.e., body sensations, “just right,” “inner tension,” and “urge only”) and a severity scale. The USP-SPS severity scale measures the severity of sensory phenomena on three 6-point anchored ordinal scales that focus on the frequency of the sensory phenomena (0 to 5), the amount of distress they cause (0 to 5), and the degree to which they interfere with patient functioning (0 to 5). The total score (ranging from 0 to 15) is obtained by combining these scores.
- The Brown Assessment of Beliefs Scale (BABS) [50]—the Portuguese version of the Brown Assessment of Beliefs Scale (BABS) was used to assess participants’ insight into several dimensions, including beliefs, perception of others’ views and explanations of different views, fixity of the belief, attempts to disprove it, insight into the cause of the belief, and presence of the idea of reference. Higher scores indicate poorer insight.

For the purpose of analysis, in this study, the term “stressful life events” (SLEs) was used to refer to the presence of any of the SLEs listed in Part I of the Yale OCD Natural History Questionnaire (Table A1), as described above. Possible answers to the questions regarding SLEs before the onset of OCD were “no,” “I don’t know,” and “yes.” Of the 1001 evaluated patients, 605 (60.5%) answered “yes” to having experienced an SLE. The remaining group of 396 patients (39.6%) was designated as the negative SLE group (i.e., the control group).

### 2.2. Statistical Analysis

Initially, the frequencies and distributions of the variables that were selected for the sample as a whole were calculated, using means (standard deviation), medians (with minimum and maximum values), and absolute (*n*) and relative (%) values. For the differences between groups, we used Pearson’s or Yates’s chi-square tests for categorical (polytomous or dichotomous) variables. Scores (on the Y-BOCS, DY-BOCS, BDI, and BAI) were analyzed as continuous variables and compared using the Student’s *t*-test or the Mann–Whitney *U* test according to the results of the Kolmogorov–Smirnov test for normal distribution. To calculate the probability of an event as a function of other events, thus reducing the “data mining” effect of exploratory studies with several variables [51,52], we employed two models of logistic regressions (probabilities for entry and removal of 0.05 and 0.10, respectively) to identify factors independently associated with SLE occurrence (dependent variable), controlling for confounding factors (sociodemographic and clinical aspects). The models were performed as follows: Model 1—to provide a better specific description of psychopathological features, a stepwise logistic regression was run for each variable of the following clinical aspects that resulted in a *p*-value lower than 0.10 after the univariate analysis: sociodemographic features (Table 1), intrinsic factors (Table 2), extrinsic factors (Table 3), and psychiatric comorbidities (Table 4); Model 2—a unique logistic regression (enter model) was run for every variable that resulted in a *p*-value lower than 0.10 after the univariate analysis, except for those that showed overfitting while performing Model 1.

For the second analysis (stepwise regression), the *p*-value was 0.05. The variance inflation factor (VIF) calculation verified that there was no relevant collinearity between continuous variables (VIF = 2.3). We considered the suggestion of Chen and colleagues that odds ratios (ORs) of 1.68, 3.47, and 6.71 are equivalent to Cohen’s *d* effect sizes of 0.2 (small), 0.5 (medium), and 0.8 (large), respectively [53]. To verify the replicability of the logistic regression models, we performed a holdout cross-validation analysis. This method is indicated when a large amount of data is available and consists of randomly dividing the total dataset into two mutually exclusive subsets, one for training with 70% of the total sample (parameter estimation) and another with the remaining 30% of the total sample for testing (validation) [54]. After partitioning, the model estimation was performed and, subsequently, the test data were applied, and the prediction error was calculated. The Pearson correlation between the calculated prediction error and the dependent variable was performed for the 70% training subset and the 30% testing subset. When the testing subset correlation coefficient was higher than that of the training subset coefficient, a non-overfitting model was considered [54]. The level of statistical significance was set at 5%. Analyses were executed with SPSS version 17.0 (SPSS Inc., Chicago, IL, USA) and Win-PEPI 11.0. (Hebrew University, Jerusalem, Israel) 

## 3. Results

### 3.1. Describing SLEs in OCD Patients

Approximately 60% of the patients (*n* = 605) reported having experienced an SLE of any type. The most common self-related lifetime SLEs were emotional problems in the family (*n* = 231; 38.2%), moving to another city/neighborhood (*n* = 210; 34.7%), family financial problems (*n* = 139; 23.0%), being in love/starting a significant relationship/marrying (*n* = 127; 21.0%), health problems in the family (*n* = 118; 19.5%), childbirth (especially the birth of a brother or sister) (*n* = 101; 16.7%), death of a relative/friend (*n* = 99; 16.4%), having a clinical disease of any type (*n* = 85; 14.0%), a significant relative moving to another city/neighborhood (*n* = 80; 13.2%), ending a significant relationship/divorcing (*n* = 60; 9.9%), and housing problems (*n* = 59; 9.8%).

### 3.2. Comparing OCD Patients Who Have or Have Not Experienced an SLE

As seen in Table 1, the SLE group appears to comprise a greater number of self-declared nonwhite skin color and socioeconomic class C (instead of class A of the negative SLE group) individuals, with a lower number of years studied and a lower mean personal income.

### 3.3. Intrinsic Psychopathological Features

Regarding the presence of specific OCS, the SLE group presented a higher prevalence of aggressive, sexual/religious, and hoarding content when compared to that of the non-SLE group (Table 2). The same occurred for all types of sensory phenomena (especially for body sensations, the “just right” sensation, and inner tension) and all types of chronic tics. Both groups presented a prevalent early onset of OCS (50.9% of the cases), but the SLE group seemed to experience a later onset of OCD symptoms (after 18 years old) when compared to the non-SLE group, while the non-SLE group more frequently experienced intermediate onset (during adolescence) than the SLE group. Table 2 also shows that, except for the “aggressive” and “contamination/washing/cleaning” dimensions, the other OCS dimensions were more severe for the SLE group. The total severity of OCD, as measured by the Y-BOCS or the DY-BOCS, was also higher for the SLE group. Concerning the total score of the Y-BOCS for statistical significance, this was probably due to the score related to compulsions.

### 3.4. Extrinsic Psychopathological Features

Although the SLE group presented a higher prevalence of benzodiazepine use, such prevalence did not reach a significant statistical level. The same occurred for the higher prevalence of hospitalization for the non-SLE group. Both *p*-values of the above-cited variables (see Table 3), however, allowed us to include them in the regression analysis (see below). No other psychiatric treatment differed between the groups. The occurrence of SLE was statistically related to lifetime suicidal ideation (nearly 40%), although it should be noted that there was also a high level of lifetime suicidal ideation in the non-SLE group (nearly 31%). The SLE group also showed a higher median severity of depressive and anxious symptoms according to the Beck’s inventories.

As can be seen in Table 4, several psychiatric comorbidities were associated with the SLE group, especially ADHD, separation anxiety disorder, major depression, dysthymia, panic with agoraphobia, panic without agoraphobia, and post-traumatic stress disorder.

### 3.5. Logistic Regression Analysis

Table 5 summarizes the results of the two logistic regression models. For Model 1, the following variables were entered: from Table 1 (sociodemographic variables)—social class, self-declared skin color, number of years studied, and personal income; from Table 2 (OCD intrinsic factors)—the presence of aggressive, sexual/religious, hoarding, and miscellaneous DY-BOCS dimensions, the presence of any type of sensory phenomenon, the presence of any kind of chronic tic and Tourette’s syndrome, the age at onset (categorical), the severity of all DY-BOCS dimensions (except for the aggressive dimension), the DY-BOCS total score, and the Y-BOCS compulsions and total scores; from Table 3 (OCD extrinsic factors)—the use of benzodiazepines, psychiatric hospitalizations, lifetime suicidal ideation, and BDI and BAI scores; from Table 4 (psychiatric comorbidities)—ADHD, separation anxiety disorder, major depression, dysthymia, panic with agoraphobia, panic without agoraphobia, and post-traumatic stress disorder. For Model 2, all of the above-mentioned variables were entered together in a unique logistic regression analysis, except for the variables of Table 1, which were overfitted according to the cross-validation analysis. Only the variables that remained for each model are shown.

The handout cross-validation showed that the variables of Table 1 were overfitted, probably because of the suppression of other variables, such as social class and personal income, which could have magnified the role of self-declared skin color in the model. Thus, such variables were not included in the Model 2 regression.

## 4. Discussion

Several variables were evaluated in this exploratory study and, although initially (in the univariate analysis), many showed differences in prevalence and severity between the groups, only six remained statistically and epidemiologically relevant after two models of logistic regression: self-declared nonwhite skin color; comorbidities with separation anxiety, ADHD, and PTSD; the presence of any sensory phenomena; lifetime psychiatric hospitalization. Despite the cross-sectional outline of this study and its reverse causality effect, it is possible that self-declared skin color (obviously) and comorbidity with separation anxiety and ADHD are conditions that occurred prior to the OCD onset, as described by de Mathis et al. [55]; in this way, they may be considered conditions of vulnerability for the sample. Otherwise, the association of SLEs and PTSD in OCD patients is fully comprehensible, since SLEs and/or traumatic events are necessary to develop diagnostic criteria for PTSD [56] (American Psychiatric Association, 2013). However, to the best of our knowledge, this is the first study to explore and find a relationship between SLEs and sensory phenomena in this population. Although psychiatric hospitalization remained a relevant factor after logistic regression, this may be due to the severity of the OCD symptoms, to the development of PTSD and its context, or to the subsequent severity of depressive symptoms at the moment of the traumatic event. It may also be a spurious result due to the large number of variables analyzed. As described in the methodology, the logistic regression retained variables with an OR lower than 1.50(such as depressive symptoms, severity of BDI, severity of sexual/religious and hoarding DY-BOCS dimensions, and early onset), which were not considered epidemiologically significant and will also not be discussed below.

### 4.1. Sociodemographic Features Related to Stressful Life Events—Self-Declared Nonwhite Skin Color

According to Lopes, Faerstein, and Chor [57], the high rates of violence and crime, unequal access to health, inadequate housing conditions, social inequality, and unemployment rate make the Brazilian population particularly susceptible to SLEs. As the majority of the population in Brazil is self-declared nonwhite (according to the Brazilian Institute of Geography and Statistics (IBGE) (2010), 52.16% self-declare to be black, yellow, “pardo,” or Indian), we could argue that Brazilian OCD patients may follow the tendency of their country and may also be more frequently exposed to SLEs, independently of skin color. Corroborating this, Medeiros et al. [58] found that Brazilian OCD patients, when compared to U.S. ones, seemed to have significantly greater rates of generalized anxiety disorder and post-traumatic stress disorder and to be less frequently identified as Caucasian [58]. Thus, this nonwhite skin color finding would just be a specific feature of our Brazilian sample. However, one point for discussion is that our whole sample had only 16.89% nonwhite patients, differing substantially from the Brazilian population census. As self-declared nonwhite people are more prone to social disadvantages in Brazil because of the historical social context (such inequalities present at different moments in an individual’s lifetime, beginning in childhood and continuing through the school years, in terms of access to urban infrastructure and crystallizing in the labor market, consequently determining the income and living conditions of Afro-Brazilians as a whole [59]), this factor, added to a psychiatric diagnosis (OCD in this case) [60], may make this subsample more prone to SLEs, which may constitute a specific vulnerable group that may need further health assistance. However, this result must be interpreted carefully, since the model was overfitted due, probably, to a suppression effect of other variables, such as social class and personal income, which could have magnified the role of self-declared skin color in the model.

### 4.2. Comorbidity with Separation Anxiety, ADHD, and PTSD

De Mathis et al. [55], when analyzing the trajectory of psychiatric comorbidities of OCD patients, described that separation anxiety and ADHD have a mean age of onset prior to OCD symptoms (6, 7, and 12 years old, respectively). Curiously, but unsurprisingly, OCD patients who presented with separation anxiety disorder as a first diagnosis had a higher lifetime frequency of post-traumatic stress disorder, a result that was not found for OCD patients who initially presented with ADHD [55]. Unfortunately, de Mathis et al. [55] did not perform a longitudinal analysis or an individual retrospective account when they developed the symptoms for each comorbid disorder; these are needed to better answer this question. Nevertheless, it may be argued that those two comorbidities may be related to SLEs in OCD patients, because patients may have become less resilient since early childhood, leading to a higher susceptibility to misfortune or change and an inability to recover or adjust easily [61,62]. As separation anxiety and ADHD may interfere with the development of attachment style, their occurrence prior to OCD may also lead patients to having lower resilience. To corroborate this argument, Zakiei et al. [63] showed a positive association between ambivalent/avoidant attachment styles and obsessive–compulsive personality disorder, as well as a negative correlation between resilience and obsessive–compulsive personality disorder [63]. 

Although the occurrence of comorbid PTSD may contribute to the presence of specific symptom content dimensions, as for the aggressive and hoarding dimensions [19], our results did not confirm our previous hypothesis, since the OR values in the logistic regression analyses were too low to be epidemiologically significant.

Perhaps the extensive heterogeneity of existing SLEs, multiplied by the different individual ways of processing the same environmental event and added to the lack of a standardized and comprehensive scale of SLEs, represent a real challenge in this field of research. As an example, Carr et al. [64] separated SLEs into subtypes (i.e., emotional abuse, physical abuse, sexual abuse, and neglect) in a systematic review that attempted to relate specific SLEs to specific mental disorders. In the present study, a patient was placed in the SLE group only because he presented an SLE listed in Part I of the Yale OCD Natural History Questionnaire. We believe that this is a limitation of our study, which prevented us from finding more credible results, at least in this respect. Regardless of this, we believe that the heterogeneity of existing SLEs should be the subject of new studies aimed at discovering the real role of their particularities.

In accordance with our results, Frías Ibáñezet al. [65] also postulated that SLEs constitute a nonspecific risk factor for OCD, either as predisposing or precipitating factors. Additionally, SLEs can determine OCD pathoplasty, reflecting the diversity of symptom presentation in patients. They also suggested a new clinical entity called “post-traumatic obsessive–compulsive disorder” [65]. Comorbid PTSD seems to be a risk factor of a lack of response to treatment in OCD patients [66], making this comorbidity an important factor in robust evidence-based therapeutic strategies.

### 4.3. Sensory Phenomena Related to Stressful Life Events

The way in which SLEs may be related to sensory phenomena in OCD patients is yetto be investigated. As sensory phenomena are robustly linked to tic-related OCD [12,17], including neurobiological distinct features when present in OCD patients (i.e., gray matter volume increases in the left sensorimotor cortex) [13], the DSM-V has recently acknowledged a new “tic-related” specifier for OCD [55]. Ferrão et al. [12] found that approximately 65% of OCD patients show at least one type of sensory phenomenon, and at least 15% describe it as more severe than the obsessions [12].

Considering sensory phenomena in OCD patients, “interoceptive awareness” could be related to the cognitive processing of emotional stimuli, improving decision-making under risk, regulating emotion, improving the acquisition of fear conditioning and implicit memory for emotional words and/or pictures, and increasing cognitive interference from negative words; it has also been shown to moderate the relationships between behavior and brain functioning and between behavior and physiology [67]. Some studies show increased physiological arousal in patients with increased anxiety [68], making it possible that an increased awareness of, or attention to, body sensations contributes to the sensory phenomena experienced in OCD [69].

Considerable research has also identified increased activation and connectivity in the insula of patients with OCD [70,71,72], making this structure a main neural node in the interception of OCD, sensory phenomena, and anxiety, especially for patients with higher anxiety levels due to SLEs [67]. Another hypothesis is that SLEs may increase anxiety levels, triggering immunological alterations that persist across time and, thereby, promote a continuous subclinical effect on inflammatory processes, reducing individuals’ immune defense and promoting a link between psychological stress and physical symptoms [73]. This process may increase the perception and/or severity of sensory phenomena, which, in turn, may lead to repetitive behaviors (including compulsive acts). Acting on these compulsions may lead to relief of the sensory phenomenon and/or anxiety, which, in turn, may increase the “need” to perform the action over and over again.

Patients with prominent anxiety symptoms, including OCD ones, generally overreact to environmental/emotional stimuli not only due to chronic increased amygdala activation [74] but also due to the connectivity between the frontostriatal system areas, especially the orbitofrontal cortex (OFC), cingulate gyrus, and basal ganglia [75]. As described by Bhikram et al. (2020) [76], sensory phenomena wereassociated with less connectivity between the OFC and sensorimotor cortex and between the inferior frontal gyrus and the putamen and insula seeds. Thus SLE, as external event (environmental/emotional) may activate or exacerbate vulnerable neurocircuits related to OCD phenomena (obsessions, compulsions, and sensoryphenomena), but this statement needs further investigation.

In our study, sensory phenomena were significantly associated with SLEs. Despite having a significant association, this result could also be caused by the fact that questions regarding sensory phenomena were frequently asked to all patients in the database, since they were being cared for in facilities specializing in OCD. In other studies regarding OCD patients in facilities not specializing in a particular disorder, such specific questions may not have been addressed.

### 4.4. Psychiatric Hospitalization

Since SLEs were significantly associated with the presence of a PTSD diagnosis in our study, we hypothesize that this can have an impact on the association of SLEs and psychiatric hospitalization as well. PTSD can be a very severe disorder and is one of the few psychological conditions that predict suicidal behavior [77]. Since many patients with PTSD present clinically with an elevated risk of suicide [77], it is predictable that there will be an increase in psychiatric hospitalization in a population with reported SLEs, which are part of a PTSD diagnosis. In fact, MacFarlane found, in a sample of 141 hospitalized psychiatric patients, that 61% reported at least one traumatic event during their lifetime and 28% met the formal DSM-III-R criteria for a lifetime diagnosis of PTSD [78]. However, the association between OCD, SLEs, and psychiatric hospitalization needs further investigation.

## 5. Conclusions

This cross-sectional study allowed us to find some interesting associations between SLEs and specific characteristics of OCD. The association between SLEs and sensory phenomena was the most innovative finding of the study, allowing us to strengthen the evidence on the effect of environmental aspects affecting the neurobiology of OCD.

Associations between skin color and separation anxiety with reported SLEs initiating or worsening OCD symptoms were found. These findings allow us to postulate that separation anxiety and skin color (nonwhite) may lead to greater vulnerability to SLEs. The neurobiological mechanism of this interaction, however, needs further analysis. An association between SLEs and PTSD comorbid with OCD was also found. This association, however, is very predictable, bearing in mind that SLEs and/or traumatic life events are necessary to fulfill the diagnostic criteria for PTSD.

Our results cannot be generalized to populations treated at the primary or secondary healthcare levels and might not have broad external validity, since our sample was obtained from centers specializing in treating OCD, which attract patients with a higher severity of symptoms.

Although the study analyzed a large sample and had an exploratory aim, our results should be interpreted with caution, since the statistical approach (i.e., stepwise logistic regression) could lead to erroneous results, overfitting the significance of the model’s remaining variables [79,80]. Other statistical strategies, such as hierarchical regressions or even machine learning strategies, may be considered in future studies.

A limitation of this study is that the time of occurrence of SLEs and the time of obsessive–compulsive symptoms onset were not compared, since we do not have data on these important aspects. As a consequence, causality between SLEs and OCD aspects in our study becomes harder to establish. This study does not compare OCD patients with the general population and its rates of SLEs because our database includes only patients with OCD seen in tertiary services. As a result, the generalization of our results should be seen with caution. Another limitation of the present study is that the data were obtained between 2003 and 2008. Even though not much has changed regarding our understanding and treatment of OCD or of the types or quality of common SLEs since then, it is possible that this may have impacted our findings, making it more difficult to generalize our results to patients with OCD in the present moment. Fortunately, a new version of the CTOC is underway to assess this issue, which will possibly be compared to the present data. 

## Figures and Tables

**Table 1:** Sociodemographic comparison between groups who have or have not experienced a stressful life event (SLE).

	SLE Group (*n* = 605)		Non-SLE Group (*n* = 396)		Statistics Test (*p*) (φ Effect Size)
	*n*	%	*n*	%	
**Female**	353	58.3	216	54.5	χ_Yates_ = 1.26 (0.26)
Marital status					χ_Pearson_ = 2.36 (0.31)
Single	238	39.3	139	35.1	
Currently with a partner	317	52.4	227	57.3	
Currently separated from partner	50	8.3	30	7.6	
**With offspring**	246	40.7	144	36.4	χ_Yates_ = 1.68 (0.20)
Have a religion	59	9.8	47	11.9	χ_Yates_ = 0.92 (0.34)
Practice the religion	334	55.2	213	53.8	χ_Yates_ = 0.00 (1.00)
**Not Working**	307	50.9	194	49.1	χ_Yates_ = 0.24 (0.62)
**Social Class (ABIPEME)**					χ_Pearson_ = 52.9 **(<0.001)** **(0.23)**
Class A	59	9.7	106	26.7	
Class B	246	40.5	139	35.1	
Class C	237	39.2	112	28.2	
Class D	47	7.7	26	6.6	
Class E	12	1.9	10	1.4	
**Self-declared skin color** **(nonwhite)**	119	19.7	50	12.6	χ_Yates_ = 7.97 **(0.005)** **(0.22)**
	**Mean**	**SD**	**Mean**	**SD**	**Statistics** **Test (*p*)** **(Cohen’s *d*)**
**Current age (years)**	35.5	12.7	34.2	13.3	*t*_Student_ = 1.47 (0.14)
**Number of years studied**	14.2	4.7	15.3	5.3	*t*_Student_ = 3.37 **(0.001)** **(0.22)**
**Personal income** **(minimum wage)**	2.8	1.2	3.3	1.3	*t*_Student_ = 5.34 **(<0.001)** **(0.40)**

SLE, stressful life event; *p*, level of statistical significance; *n*, absolute value; %, relative value; χ_Yates_, Yates’s chi-square; χ_Pearson_, Pearson’s chi-square; ABIPEME, socioeconomic classification of the AssociaçãoBrasileira de Pesquisa de Mercado; SD, standard deviation; *t*_Student_, Student’s *t*-test. φ effect size and Cohen’s *d* were calculated only for those variables with *p* < 0.05. All significant effects have been bolded.

**Table 2:** Comparison of the intrinsic features of psychopathological obsessive–compulsive disorder (OCD) between the SLE and non-SLE groups.

	SLE Group (*n* = 605)		Non-SLE Group (*n* = 396)		Statistics Test (*p*) (φ Effect Size)
	*n*	%	*n*	%	
**Presence of DY-BOCS dimensions**					
**Aggressive**	433	71.6	239	60.4	χ_Yates_ = 13.1 **(<0.001) (0.14)**
**Sexual/religious**	378	62.5	194	49	χ_Yates_ = 17.2 **(<0.001) (0.17)**
**Symmetry/ordering**	531	87.8	337	85.3	χ_Yates_ = 1.05 (0.31)
**Contamination/washing**	451	74.5	286	72.2	χ_Yates_ = 0.55 (0.46)
**Hoarding**	342	56.5	186	47	χ_Yates_ = 8.4 **(0.004) (0.13)**
**Miscellaneous**	538	88.9	335	84.6	χ_Yates_ = 3.64 (0.056)
**Sensory phenomena (any type)**	410	67.8	241	60.9	χ_Yates_ = 4.73 **(0.03) (0.09)**
Body sensations	259	42.8	112	28.3	χ_Yates_ = 21.0 **(<0.001) (0.24)**
“Just right” sensations	338	55.9	181	45.7	χ_Yates_ = 9.5 **(0.002) (0.14)**
Incompleteness	114	18.8	62	15.7	χ_Yates_ = 1.46 (0.23)
Inner tension	99	16.4	45	11.4	χ_Yates_ = 4.46 **(0.035) (0.18)**
“Urge” sensations	147	24.3	93	23.5	χ_Yates_ = 0.05 (0.83)
**Chronic tics (any type)**	192	31.7	92	23.2	χ_Yates_ = 8.1 **(0.004) (0.17)**
**Tourette’s syndrome**	62	10.2	26	6.6	**χ_Yates_ = 3.6 (0.058) (0.20)**
**Age at onset (categorical)**					χ_Pearson_ = 10.3 **(0.006) (0.10)**
Early (<11 years old)	300	50.9	190	50.9	
Intermediate (11–18)	191	32.4	146	39.1	
Late (≥18 years old)	98	16.6	37	9	
**OCS course**	(*n* = 532)			(*n* = 359)	χ_Pearson_ = 0.09 (0.96)
Chronic/continuous	38	7.2	24	6.7	
Episodic/oscillating	263	49.4	180	50.1	
Progressively worsening	231	43.4	155	43.2	
**Familial OCD**	302	49.9	201	50.9	χ_Yates_ = 0.06 (0.81)
**Familial tics**	113	19.9	79	20.9	χ_Yates_ = 0.08 (0.78)
	**Mean**	**SD**	**Mean**	**SD**	
**OCS duration (in years)**	22.7	13.2	22	13.2	*t*_Student_ = 0.77 (0.44)
	**Median**	**(Min**–**Max)**	**Median**	**(Min**–**Max)**	**Statistics**
					**Test (** ***p*)**
					**(Glass effect size)**
**Severity of DY-BOCS dimensions**					
Aggressive	9	(1–15)	9	(1–15)	U_MW_ = 42,263.5 (0.38)
Sexual/Religious	9	(1–15)	8	(1–15)	U_MW_ = 25,901.5 **(0.04) (0.78)**
Symmetry/ordering/arrangement	10	(1–15)	9	(1–15)	U_MW_ = 73,878.5 **(0.04) (0.38)**
Contamination/washing/cleaning	10	(1–15)	9	(1–15)	**U_MW_ = 49**,**696.5 (0.07) (0.58)**
Hoarding	7	(1–15)	6	(1–15)	U_MW_ = 21,537.5 **(0.006) (0.82)**
Miscellaneous	10	(1–15)	9	(1–15)	U_MW_ = 75,618.5 **(0.049) (0.37)**
**DY-BOCS total score**	23	(2–30)	22	(1–30)	U_MW_ = 101,558 **(<0.001) (0.15)**
**Y-BOCS**					
Obsessions score	13	(1–20)	13	(3–20)	U_MW_ = 109,860.0 (0.12)
Compulsions score	13	(3–20)	13	(2–20)	U_MW_ = 108,163.5 **(0.048) (0.10)**
**Y-BOCs total score**	26	(7–40)	25	(7–40)	U_MW_ = 108,143.5 **(0.024) (0.10)**
**Age at OCS onset**	10	(3–50)	10	(3–54)	U_MW_ = 104,221.0 (0.23)
**Gap of time without treatment**	15	(0–56)	14	(0–58)	U_MW_ = 102,008.5 (0.93)
**USP-SPS total score**	8	(1–15)	7	(1–15)	U_MW_ = 42,103.5 (0.25)
**BABS**	6	(0–24)	6	(0–24)	U_MW_ = 105,267.5 (0.12)

OCD, obsessive–compulsive disorder; SLE, stressful life event; *p*, level of statistical significance; *n*, absolute values; %, relative values; DY-BOCS, Dimensional Yale–Brown Obsessive–Compulsive Disorder Scale; Y-BOCS, Yale–Brown Obsessive–Compulsive Disorder Scale; USP-SPS, University of São Paulo Sensory Phenomena Scale; BABS, Brown Assessment of Beliefs Scale; χ_Yates_, Yates’s chi-square; χ_Pearson_, Pearson’s chi-square; OCS, obsessive–compulsive symptoms; Min, minimum value; Max, maximum value; U_MW_, Mann–Whitney *U* test. φ effect size and Glass effect size were calculated only for those variables with *p* < 0.05. All significant effects have been bolded.

**Table 3:** Comparison of the extrinsic features of psychopathological OCD between the SLE and non-SLE groups.

	SLE Group (*n* = 605)		Non-SLE Group (*n* = 396)		Statistics Test (*p*) (φ Effect Size)
	*n*	%	*n*	%	
**Any previous psychiatric treatment**	468	77.4	294	74.2	χ_Yates_ = 1.11 (0.29)
SSRI	431	71.2	277	69.9	χ_Yates_ = 0.14 (0.71)
Clomipramine	202	33.4	120	30.3	χ_Yates_ = 0.91 (0.34)
Other antidepressants	86	14.2	55	13.9	χ_Yates_ = 0.003 (0.96)
Benzodiazepines	243	40.2	135	34.1	χ_Yates_ = 3.50 **(0.06) (0.10)**
Mood stabilizers	63	10.4	44	11.1	χ_Yates_ = 0.06 (0.81)
Lithium	32	5.3	17	4.3	χ_Yates_ = 0.32 (0.57)
Neuroleptics	119	19.7	84	21.2	χ_Yates_ = 0.26 (0.61)
**Any previous psychotherapy**	389	64.3	254	64.1	χ_Yates_ = 0.00 (1.00)
CBT (E/RP)	93	23.9	63	24.8	χ_Yates_ = 0.03 (0.87)
**Psychiatric hospitalization**	33	5.5	35	8.8	χ_Yates_ = 3.81 (0.051)
**Suicidality**					
Suicidal ideation—lifetime	232	39.7	116	30.9	χ_Yates_ = 7.26 (0.007)
Suicidal ideation—current	71	12.2	33	8.8	χ_Yates_ = 2.32 (0.13)
Suicide attempt	71	12.2	33	8.8	χ_Yates_ = 2.32 (0.13)
Familial history of suicide	101	17.3	55	14.7	χ_Yates_ = 0.94 (0.33)
	**Median**	**(Min–Max)**	**Median**	**(Min–Max)**	**Statistics** **Test (*p*)** **(Glass effect size)**
**Beck Depression Inventory**	17	(0–52)	13	(0–53)	U_MW_ = 97,271.0 **(<0.001) (0.19)**
**Beck Anxiety Inventory**	16	(0–52)	12	(0–53)	U_MW_ = 97,590.0 **(<0.001)** **(0.18)**

OCD, obsessive–compulsive disorder; SLE, stressful life event; *p*, level of statistical significance; *n*, absolute values; %, relative values; χ_Yates_, Yates’s chi-square; SSRI, selective serotonin reuptake inhibitor; CBT (E/RP), cognitive behavioral therapy (exposition/response prevention); Min, minimum value; Max, maximum value; U_MW_, Mann–Whitney *U* test.φ effect size and Glass effect size were calculated only for those variables with *p* < 0.05. All significant effects have been bolded.

**Table 4:** Comparison of psychiatric comorbidities between SLE and non-SLE groups.

	SLE (*n* = 605)		Non-SLE (*n* = 396)		Statistics Test (*p*) (φ Effect Size)
	*n*	%	*n*	%	
**ADHD**	95	15.7	42	10.6	**χ_Yates_ = 4.84 (0.028) (0.19)**
Separation anxiety disorder	37	6.1	12	3.0	**χ_Yates_ = 4.28 (0.039) (0.29)**
Major depression	220	36.4	112	28.3	**χ_Yates_ = 6.69 (0.01) (0.14)**
Dysthymia	78	12.9	34	8.6	**χ_Yates_ = 4.05 (0.044) (0.19)**
Affective bipolar disorder	16	2.6	7	1.8	χ_Yates_ = 0.48 (0.46)
Alcohol use disorder	31	5.1	14	3.5	χ_Yates_ = 1.06 (0.30)
Panic + agoraphobia	49	8.1	20	5.1	χ_Yates_ = 3.01 (0.08)
Panic	34	5.6	11	2.8	**χ_Yates_ = 3.87 (0.049) (0.29)**
Agoraphobia	29	4.8	17	4.3	χ_Yates_ = 0.05 (0.83)
Social anxiety	198	32.7	122	30.8	χ_Yates_ = 0.32 (0.57)
Simple phobia	190	31.4	118	29.8	χ_Yates_ = 0.22 (0.64)
PTSD	82	13.6	17	4.3	χ_Yates_ = 22.0 **(<0.001) (0.47)**
Generalized anxiety disorder	213	35.2	125	31.6	χ_Yates_ = 1.26 (0.26)
Somatization disorder	16	2.6	7	1.8	χ_Yates_ = 0.48 (0.49)
Pain disorder	20	3.3	7	1.8	χ_Yates_ = 1.61 (0.20)
Hypochondria	18	3.0	14	3.5	χ_Yates_ = 0.89 (0.64)
Body dysmorphic disorder	72	11.9	39	9.8	χ_Yates_ = 2.25 (0.28)
Any eating disorder	51	8.4	27	6.8	χ_Yates_ = 0.66 (0.42)
Grooming behavior disorder	128	21.8	76	19.2	χ_Yates_ = 0.46 (0.50)
Intermittent explosive disorder	39	6.5	20	5.1	χ_Yates_ = 0.57 (0.45)
Compulsive buying disorder	52	8.6	23	5.9	χ_Yates_ = 2.21 (0.14)
Compulsive sexual behavior	15	2.5	6	1.6	χ_Yates_ = 0.64 (0.42)
Internet addiction	15	2.5	8	2.0	χ_Yates_ = 2.07 (0.36)

SLE, stressful life event; *p*, level of statistical significance; *n*, absolute values; %, relative values; χ_Yates_, Yates’s chi-square; ADHD, attention-deficit/hyperactivity disorder; PTSD, post-traumatic stress disorder.φ effect size was calculated only for those variables with *p* < 0.05. All significant effects have been bolded.

**Table 5:** Logistic regression models for the variables with *p* < 0.10 in the univariate analysis.

Model 1—Table-by-Table Analysis (forward Conditional Method)			Cross-Validation Analysis		
**Variable that remained in the model**	***p***	**OR** **(95% CI)**	**Training Subset** **(70%)**	**Testing** **Subset** **(30%)**	**Conclusion**
**Table 1—sociodemographic variables**			**r_P_(*p*)**	**r_P_(*p*)**	
Self-declared skin color (nonwhite)	0.033	1.51 (1.18–2.42)	0.176 (<0.001)	0.095 (0.104)	Overfitted
**Table 2—OCD intrinsic factors**					
Presence of sensory phenomena	<0.001	1.47 (1.04–1.76)	0.193 (<0.001)	0.208 (<0.001)	Not overfitted
Severity of sexual/religious DY-BOCS dimension	<0.001	1.06			
Severity of hoarding DY-BOCS dimension	0.001	1.06			
**Table 3—OCD extrinsic factors**					
BDI (depression severity)	<0.001	1.03	0.131 (0.001)	0.168 (0.003)	Not overfitted
**Table 4—psychiatric comorbidities**					
ADHD	0.042	1.18 (1.07–2.31)	0.147 (<0.001)	0.149 (0.01)	Not overfitted
Separation anxiety	0.037	1.31 (1.07–4.05)			
PTSD	<0.001	1.42 (1.04–4.99)			
**Model 2—analysis of all variables together (enter method)**					
**Variable that remained in the model**	***p***	**OR** **(CI 95%)**	**Training Subset** **(70%)**	**Testing** **Subset** **(30%)**	**Conclusion**
			**r_P_(*p*)**	**r_P_(*p*)**	
Early onset	0.003	1.03 (1.01–1.05)	0.208 (<0.001)	0.214 (<0.001)	Not overfitted
Severity of hoarding DY-BOCS dimension	0.005	1.05 (1.02–1.09)			
Severity of sexual/religious DY-BOCS dimension	0.006	1.04 (1.01–1.07)			
Psychiatric hospitalization	0.015	1.95 (1.14–3.35)			
Beck Depression Inventory score	0.04	1.01 (1.00–1.03)			
PTSD	<0.001	2.93 (1.62–5.29)			
Presence of sensory phenomena	<0.001	1.87 (1.39–2.52)			

*p*, level of statistical significance; OR, odds ratio; r_P_, Pearson correlation coefficient; OCD, obsessive–compulsive disorder; DY-BOCS, Dimensional Yale–Brown Obsessive–Compulsive Scale; BDI, Beck Depression Inventory; ADHD, attention-deficit/hyperactivity disorder; PTSD, post-traumatic stress disorder. Overfitting occurred when the training subset r_P_ was higher than the testing subset r_P_; while when the training subset r_P_ was lower than the testing subset r_P_, overfitting did not occur.

## Appendix A

**Table A1:** List of stressful life events (SLEs) extracted from the Yale OCD Natural History Questionnaire.

List of SLEs
Emotional problems in the family
Moving to another city/neighborhood
Relative moving to another city/neighborhood
Family with financial problems
Being in love/starting a significant relationship/marrying
Health problems in the family
Pregnancy (own)
Pregnancy (of spouse/girlfriend/partner)
Childbirth (own child)
Childbirth (child of a relative)
Death of a relative/friend
Having any type of clinical disease
Significant relative moving to another city/neighborhood
Ending a significant relationship/divorcing
Family with housing problems
Started using illicit drugs
Family with legal problems
